# Supplementary figures and images for: Geographic Range Overlap Rather than Phylogenetic Distance Explains Rabies Virus Transmission among Closely Related Bat Species
Source: Viruses. 2022 Oct 29;14(11):2399. doi: 10.3390/v14112399 (PMC9697534; doi:10.3390/v14112399)

**A**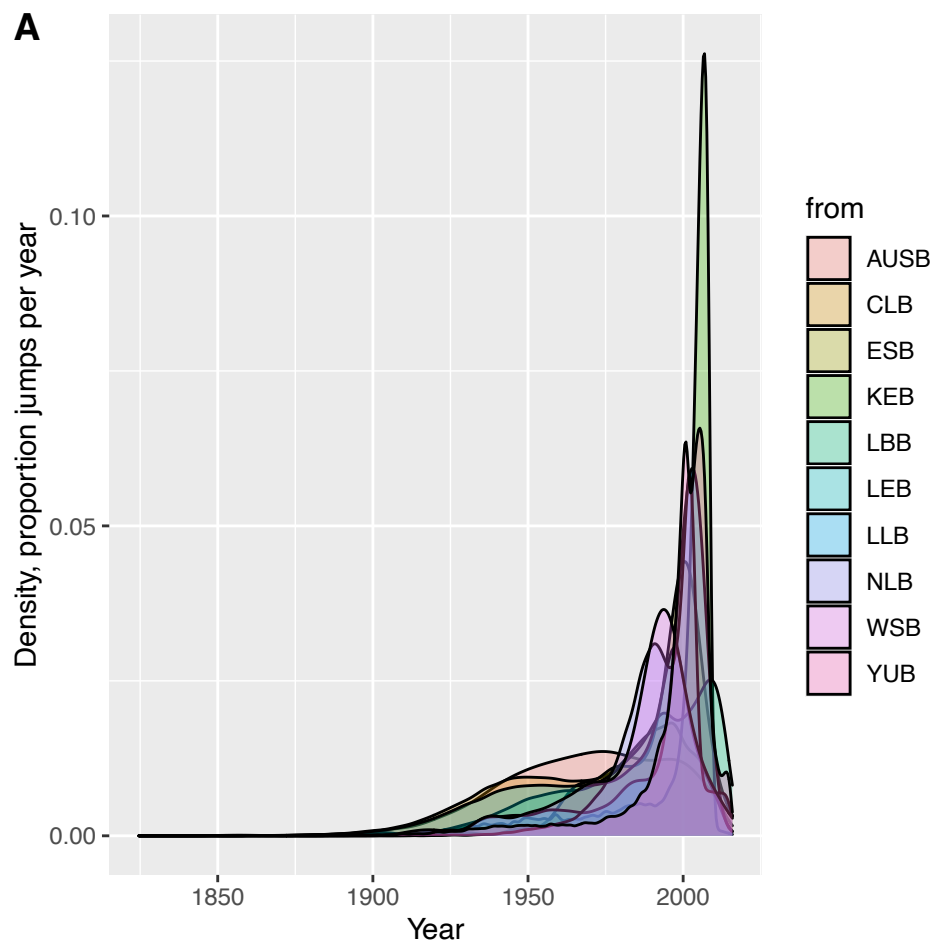**B**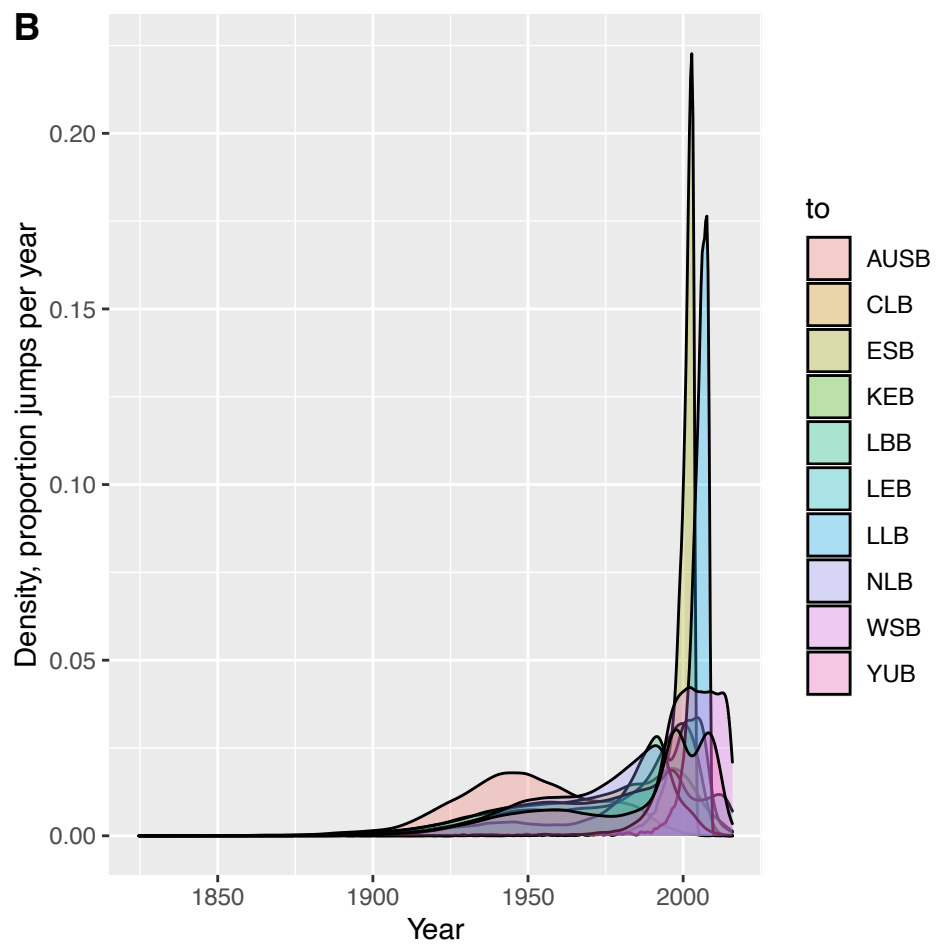

Supplement: Supplementary file 1 [file viruses-14-02399-s001.zip › Figure S1.pdf]

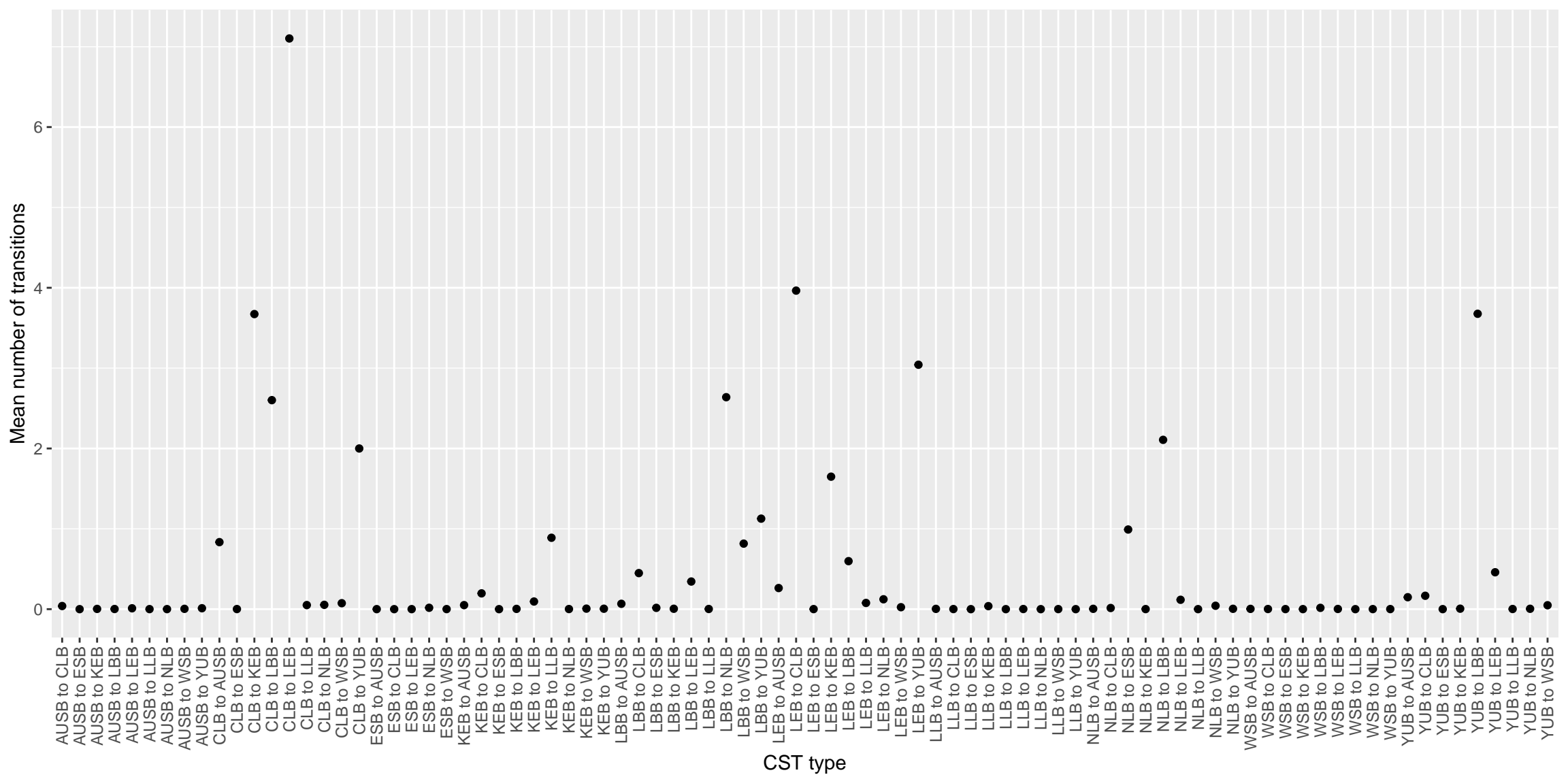

Supplement: Supplementary file 1 [file viruses-14-02399-s001.zip › Figure S2.pdf]
